# Modern Assisted Reproductive Technologies and Bioethics in the Islamic Context

**DOI:** 10.1080/14746700.2021.1910914

**Published:** 2021-04-19

**Authors:** Mansooreh Saniei, Mehdi Kargar

**Keywords:** Assisted reproductive technologies, third-party donation, surrogacy, sex selection, bioethics, *Shi’a*, *Sunni*, Islam

## Abstract

During the last few decades, infertility has been discussed as a socio-cultural and medical dilemma. Infertile couples attempt to overcome this problem, including using assisted reproductive technologies (ARTs). Similar to other groups, Muslims struggle with various aspects of infertility and its treatments, trying to reconcile the use of ARTs with the regulations in respect of the socio-cultural, legal, ethical, economic, and political factors of their community. Religion usually plays a significant role in the governance of medically assisted reproduction. This paper describes the Islamic intellectuals' permissive and restrictive opinions on modern ARTs and ethics in the Islamic context.

Having children is a strong natural human desire; and infertility can pose an emotionally hard dilemma. Therefore people try to overcome this difficulty regardless of time, place, culture, religion, society, and economic situation, etc. Infertility can be described as the failure of a couple to conceive a baby after one year of unprotected, regular sexual intercourse.^1^ This worldwide issue affects 10%–15% of couples. Infertility affects men and women more or less equally; however, society tends to lay the blame on the woman regardless of the causes.^2^ Since the birth of Louise Brown, the world’s first test-tube baby in the United Kingdom, 1978, assisted reproductive technology (ART) has made great progress and is responsible for the birth of “more than eight million” babies.^3^

Over the last 40 years, human societies have been taking advantage of these new innovations in the areas of biomedical therapy and research, however, many methods of reproductive assistance are the cause for a number of ethical, social, and legal controversies, and this “new medicine calls all in doubt.”^4^ Hence, bioethical discourses are required when the direction of bioscience and technology is unclear. In other words, such modern innovative medicine must be approached along adequate moral guidelines and from various angles. In fact, some new innovations in science and technology, such as the new ART and/or human embryonic stem cell science, have created unique religious challenges and bioethical concepts, particularly concerning the moral status of human embryos, notions of kinship and family, women’s rights, and so on.^5^

During the last four decades, ARTs have become widespread around the globe, including Muslim countries that accept the practice of some forms of assisted reproduction, while refusing others. Islam recognises infertility as a crucial difficulty and encourages its believers to pursue legal treatment, as it would with any type of illness.^6^ Therefore, if any form of ART is accepted as a lawful and ethical cure in the Islamic context, Muslims are allowed to make use of it. The main goal of this paper is to indicate the philosophical foundation of the Islamic viewpoints as expressed on the bioethical discourses of new ARTs, such as third-party sperm and egg donation, embryo donation, surrogacy, and sex selection.

## Modern ARTs, Ethics, and Islam

### Sources and Methods in Islam

Islamic teachings provide a wide way of life for Muslims, including all aspects of individual, social, spiritual, material, and moral life and also national, international, political, and economic programmes.^7^ In fact, Islamic law governs every facet of its believers’ lives, including emerging biomedical technologies such as ARTs and/or stem cell science. Furthermore, some Muslim intellectuals observed that bioethical discussions and religion are intertwined, and ethics in the Islamic context is defined in the light of Islamic law.^8^ Accordingly, Islamic moral deliberations are based on various sources.

For the *Sunni* sect, the *holy Qur’an* (the Muslims’ holy book), *Sunnah* (the Prophet’s narrations and life), and *hadiths* (the Prophet’s sayings and actions) are fundamental sources for religious intellectuals to ratiocinate what is permitted and prohibited in Islam. The *Shi’a* sect also turns to these main sources for discussing moral issues. *Shi’a* scholars utilise the wider collection of *hadiths*, including narratives from the *Shi’a Imams* (the Prophet’s successors). In addition, for the *Shi’a*, *ijtihad* (a methodology of reasoning and an independent interpretation of a matter in hand) and *‘aql* (reason) are applied for moral deliberations and to generate *fatwas* (religious decrees).^9^ The *fatwas* instruct the followers of various religious intellectuals and authorities in how to live as good Muslims. In *Shi’a*, only high-ranking religious intellectuals (*Ayatollahs*) can issue *fatwas*, and its followers draw upon *ijtihad* and *‘aql* in their everyday lives, while the *Sunni* sect urges followers to read Islamic teachings more strictly.^10^

These various views and plans within Islamic sects have a close relationship with policy approaches to biomedical science, technology, and health, including ARTs. Local and national religious intellectuals interpret Islamic principles to determine Islamic law and to issue *fatwas*. It is noteworthy that there is no central institution to regulate bioscience and technology in the Muslim world; instead, certain organisations play the role of facilitators in this process for religious intellectuals, such as two leading organisations for the *Sunni* sects: the *Mojamaa Al-Behooth Al-Islamaya* (Islamic Research Foundation) and *Dar Al-Iftah* (House of Fatwas) in Egypt; and several associations for *Shi’a* in Qom, Shiraz, and Mashhad in Iran, and Karbala and Najaf in Iraq.^11^ In fact, religious decrees determine the bioethical guidelines for medical science, research, and technology, and then suggest a broad spectrum of explanations for the concepts of family, procreation, beginning of life, women’s rights, and other biomedical and health policies.^12^ All Islamic sects similarly choose their sources and methods of inspiration and emulation based upon their closeness to a particular religious intellectuals’ interpretation and Islamic philosophy. Accordingly, all Muslims’ sects essentially follow the same approaches to the process of reasoning for accepting, adapting, or rejecting science and technologies; nonetheless, Islam does not offer exact statements of particulars for biomedical scientific practice and research.^13^ Thus, the integration of moral principles and bio-scientific innovations are different among Islamic sects and from one Muslim society to another, as well as within sects’ diverse subsections and branches, such as *Athna Ashariah* (Twelver), *Ismaeili*, and *Zaidi* (Fiver) in the *Shi’a* sect; and *Ash’ari*, *Shafei*, *Maleki, Hanafi* in the *Sunni* school of thought.^14^

### Infertility and Reproductive Assistance

Reproductive assistance is currently used for various issues surrounding fertility, such as treating couples with infertility problems, selecting the sex of babies for fertile couples, avoiding genetic diseases through pre-implantation genetic diagnosis, helping same-sex couples to have children either by one of the partners or through third-party donors, etc.^15^

In Islam, the institution of marriage, family, and reproduction are very important. In respect to reproduction, the holy *Qur’an* states:

O Mankind! Be conscious of your Sustainer, who has created you out of one living entity, and out of it created its mate, and out of the two spread abroad a multitude of men and women. And remain conscious of God, in whose name you demand [your rights] from one another, and of these ties of kinship. Verily, God is ever watchful over you!^16^

Nonetheless, the holy *Qur’an* draws attention to the issue of infertility, and Islam advises seeking treatment for every illness, as the Muslims’ Prophet noted that God has created a remedy for every disease. Some treatments are known, others are not.^17^ As a result, a couple can attempt to get over the problem of infertility without breaching Islamic law; including the use of ARTs by a married couple. However, a woman can only utilise her husband’s sperm for fertilisation, and she is banned from the use of his gamete after divorce or his death. The babies resulting from ARTs also have some rights, such as being the authentic offspring of legitimate couples and legally inheriting the property of their parents. In this respect, in-vitro fertilisation (IVF) treatment is morally acceptable in Islam only if it is done between legal wife and husband;^18^ and modern ARTs including cryopreservation, third-party gametes (egg and sperm) donation, embryo donation, sex selection, and surrogacy might be practiced in accordance with clear ethical, social, and legal guidelines, which are based on religious beliefs and approved by relevant authorities in various Muslim societies.^19^

### Modern ARTs in the Islamic Sunni Sect

In *Sunni* schools of thought, cryopreservation is allowed only when the frozen embryos or gametes are utilised by the couple who are the biological source of the tissue, and in the context of a valid marriage. Cryopreservation is mainly applied for two reasons: (1) when ARTs lead to the generation of surplus gametes or embryos, and the couples plan to use them for further infertility treatment; (2) when someone might be affected by certain treatments such as radiotherapy or chemotherapy, which might cause infertility. In addition, this method of ARTs reduces the need for repetition of additional drug stimulation cycles and the adverse consequences of these medicines.^20^ This technique helps to preserve gametes and an embryo at its early stage of development for further utilisation by the intended parents. In case the couples do not use their frozen embryos and gametes, they are allowed to donate them to biomedical research, in accord with the proper ethical guidelines.^21^ In Islamic tradition, the beginning of life is thought to be 40–120 days after the moment of fertilisation, when the soul meets the body, which is called “ensoulment.”^22^ Therefore, the utilisation and destruction of an embryo during scientific research is not seen as murder because the embryo at its early stage of development is not considered a human being.^23^ According to the *fatwa* issued at the 17th meeting of Islamic Jurisprudence Council, the Islamic World League in Macca, Saudi Arabia:

It is allowed to isolate stem cells, to be grown and utilised for scientific research and therapy, if the source [of those cells] is legitimate, for instance, surplus fertilised eggs remaining from IVF [cycle], if donated by the couples, when it is ascertained that they will not be used in an illegal pregnancy.^24^

The Islamic *Sunni* sect does not accept the engagement of a third-party gamete, embryo, and/or womb provider for an infertile couple; and they draw analogies, for instance, between gamete donation and adultery. Islamic scholars may utilise the method of *Qiyas*, which draws analogies between the original matter and a new matter. Accordingly, the latter case has the same consequences as the former one. This method of reasoning is used when the holy *Qur’an* and *sunnah* do not directly express the constitutions which drive the *Sunni* ethical, legal, and social codes of practice.^25^ In this respect, as Marcia Inhorn^26^ observed, several religious decrees or *fatwa*s—in which they are mainly considered as the ethical regulations among Muslims—have been issued by *Sunni* institutions in the United Arabic Emirates, Qatar, Kuwait, and Saudi Arabia since 1980. These *fatwas* mostly supported IVF treatment, while prohibiting third-party ARTs in those Muslim societies.^27^ Moreover, in the late 1990s, at the ninth session of Islamic Organisation for Medical Science in Morocco, *Sunni* scholars developed an ethical, social, and legal declaration to ban human cloning and all forms of third-party ARTs which might breach the lineage of parenthood.^28^ This bioethical statement was presented to be followed by all *Sunni* sects, representing 90% of Muslims around the globe.

Surrogacy, or third-party uterus donation, is another method of ARTs which has two forms: complete and partial. In partial surrogacy (also known as straight surrogacy), an infertile couple commission a woman to donate her eggs to be fertilised by the sperm of the intended father, and then carries and gives birth to a baby for that couple. Complete surrogacy (also known as gestational surrogacy) is when the IVF embryos are generated from the sperm of the intended father and eggs of the intended mother, or a donor, and then implanted into another woman’s uterus. Therefore, unless the third party provides both the egg and uterus, there is no genetic relationship between the surrogate and the baby.^29^ *Sunni* scholars believe that Islamic law forbids surrogacy, as this method of ART causes the confusion of lineage and violates the rules of kinship. Based on the Council of the Islamic World League in Macca, surrogacy between the wives of one husband is permitted. Nonetheless, if a couple makes use of a surrogate, Muslim intellectuals made a consensus that the birth mother is the actual mother.^30^

### Modern ARTs in the Islamic *Shi’a* Sect

Almost 10%–20% of Muslims in the world are *Shi’a*, the majority of them (90%) living in Iran and parts of Bahrain, Lebanon, Iraq, Afghanistan, India, and Pakistan.^31^ Many *Shi’a* Muslims hold a liberal view towards modern ARTs and allow a certain flexibility and pragmatism towards third-party egg, sperm, embryo and uterus donation.^32^ In Iran, Ayatollah Khamenei, the supreme leader, decreed a positive *fatwa* in the late 1990s and allowed infertile couples to use all forms of donor technologies.^33^ Later, in 2002, Iran’s Islamic Consultative Assembly authorised this *fatwa* as a rule. In spite of the supreme leader’s permission and the official rule, as Morgan Clarke^34^ stated, the utilisation of modern ARTs by *Shi’a* in Iran (and elsewhere) is much more complex in reality. In fact, *Shi’a* intellectuals employ a type of individual religious reasoning, known as *ijtihad*, while *Sunni* authorities make decisions based on scriptural sources rather than individual reasoning.^35^ Therefore, this *Shi’a* mechanism of consensus fundamentally led to large heterogeneity of perspectives and practices of ARTs within *Shi’a* society. Furthermore, *Shi’a* permits a type of temporary marriage (*mut’a* or *sigheh*) which is not authorised by the *Sunni* faith. The *sigheh* is an alliance and contract between an unmarried or married Muslim man and an unmarried, divorced, or widowed Muslim woman for a fixed time period. In this frame, gamete donation is permitted through *mut’a* marriage. This method is used in Iran and in other parts of *Shi’a* communities.^36^

In the frame of *ijtihad* and *mut’a*, various views on donor technologies have emerged in *Shi’a* communities, and different *Shi’a* intellectuals have considered the permissibility of ARTs and donor technologies. Some hold the same view as the majority of *Sunnis* and ban sperm and egg donation for their followers, while others affirm that this technology is permitted under specific circumstances. Some groups of *Shi’a* scholars advise their followers to practice *mut’a* in order to make egg donation legitimate. Accordingly, the husband should temporarily marry the egg donor for a certain period of time, for instance during the period of fertilising eggs with his sperm and transferring the fertilised egg into the womb (either of his wife or a surrogate). Therefore, the infertile couple and the egg donor avoid the issue of adultery.^37^ In his permissive *fatwa*, Ayatollah Khamenei apparently expresses that for gamete donation, temporary marriage is not required, as he presumes that adultery requires the act of intercourse. In this context, even a married woman can donate her eggs to others. However, several *Shi’a* scholars, such as Ayatollah Fadlallah (a famous Lebanese *Shi’a* jurist and authority) did not approve all forms of third-party donation, for instance, he believed that a married woman cannot receive sperm from other men.^38^ And of course, a married woman is not allowed to marry another man at the same time, so she cannot practice *mut’a* with a sperm donor in order to procure a legitimate donation. Ayatollah Sistani and Ayatollah Hakim, the grand *Shi’a* religious intellectuals in Iraq, advise caution in practicing third-party donation, considering them as mostly unacceptable actions.^39^

## Sex Selection, Ethics and Islam

A large number of couples express their preference to know the gender of their babies during pregnancy. Various methods are utilised to find out the sex of foetuses, such as foetal DNA testing, chromosome analysis, sonographic tests, etc.^40^ The strong socio-cultural desire for a baby boy has led to noticeable gender ratio discrepancies in some nations including Arab states, India, China, and so on. The reason behind this desire may be expectations that male children can support family and parents economically and physically, preserve wealth and properties inside the family, maintain the family name, and/or preserve certain religious customs and traditions.^41^ In some countries, for instance in China, a broad range of gender-selective abortion had been practiced over the last four decades, which steered this society towards the serious disproportion in the gender ratio at birth and led to the issue of the “female deficit.”^42^

The utilisation of sex-selection science and technology has led to broad discussions among various disciplines including biomedicine, bioethics, philosophy, sociology, politics, law, etc. Gender selection is morally permissible when there are genetic and medical reasons; nonetheless, due to the imbalanced distribution of the gender ratio, and/or exacerbation of sex discrimination, most countries forbid the utilisation of sex selection for social and ethical reasons.^43^ In fact, the use of gender-selective technologies for non-medical reasons is considered as sex discriminatory, and many human rights organisations and bioethics institutions address this practice as an unethical, unacceptable action.^44^

In Islam, the determination and selection of gender is solely in the power of God, as the holy *Qur’an* notes that “He [God] creates what He wills. He gives to whom He wills female [children], and He gives to whom He wills males.”^45^ Accordingly, we can safely say that sex selection as a concept might represent an unacceptable intervention in the validity of Islamic law and the interruption of the divine analytical orders. There is a history of infanticide in some areas of the world, which was used as a means of gender selection. More than fourteen centuries ago, Arabs used to kill their daughters and hold their male children sacred. Islam condemned this practice and stated that on Judgment Day, “And when the girl [who was] buried alive is asked. For what sin she was killed?.”^46^ For Muslims, the use of this technology might be discrimination against females and result in prejudice and the social devaluation of women; therefore Islam condemns this technique. However, the application of technologies for sex selection in the case of a clear medical reason is acceptable.^47^

## Conclusion

Most Muslim schools of thought, *Shi’a* and *Sunni*, hold similar views on the importance of fertility, having children, infertility treatments, and IVF and modern ARTs, such as third-party donation, surrogacy, human cloning, and sex selection. In fact, Islam encourages its followers to seek treatment for infertility. IVF treatment involving the sperm and egg of a legitimate couple and the transfer of the resulting embryo(s) into the wife’s womb is clearly allowed. However, when the discussion come to the subject of donor technologies and other ART methods, there are partially divergent opinions among both Islamic sects.

The *Sunni* sect bans all forms of third-party egg, sperm, or embryo donation due to the matter of kinship and lineage. Surrogacy also is not permitted in this sect. Moreover, the supernumerary IVF embryos can be either frozen for further infertility treatment solely for the use of the contributors of those embryos and be transferred only into the same wife; or donated for research and/or destroyed. Sex selection is absolutely unacceptable, unless there is a medical reason.

In the *Shi’a* sect, many religious intellectuals allow their followers to apply for all forms of modern ARTs, except human cloning. *Shi’a* Muslims are allowed to utilise third-party gametes, embryo donation and surrogacy with or without a temporary marriage or *mut’a*. Sex selection is usually forbidden for non-medical and social reasons, but it is practiced for medical reasons.

## Notes

1 F. Zegers-Hochschild, G. Adamson, J. deMouzon, O. Ishihara, R. Mansour, K. Nygren, E. Sullivan and S. Vanderpoel, “For ICMART and WHO. International Committee for Monitoring Assisted Reproductive Technology (ICMART) and the World Health Organisation (WHO) Revised Glossary of ART Terminology,” *Fertility and Sterility* 92 (2009), 1520–1524.

2 M. N. Mascarenhas, S. R. Flaxman, T. Boerma, S. Vanderpoel and G. A. Stevens, “National, Regional, and Global Trends in Infertility Prevalence Since 1990: A Systematic Analysis of 277 Health Surveys,” *PLoS Medicine* 9 (2012), e1001356; A. Agarwal, A. Mulgund, A. Hamada and M. R. Chyatte, “A Unique View on Male Infertility Around the Globe,” *Reproductive Biology and Endocrinology* 13 (2015), 37.

3 J. Datta, M. J. Palmer, C. Tanton, L. J. Gibson, K. G. Jones, W. Macdowall, A. Glasier, P. Sonnenberg, N. Field, C. H. Mercer, A. M. Johnson and K. Wellings, “Prevalence of Infertility and Help Seeking Among 15000 Women and Men,” *Human Reproduction* 31:9 (2016), 2108–2118.

4 S. Islam, “Ethics of Assisted Reproductive Medicine: A Comparative Study of Western and lslamic Bioethics,” *International Institute of Islamic Thought (IIIT)* (2013).

5 M. Saniei and H. Baharvand, “Human Embryonic Stem Cell Science in Muslim Context: ‘Ethics of Human Dignity’ and ‘Ethics of Healing,’” *Advances in Medical Ethics* 4:1 (2018), 7–21.

6 Islam, “Ethics of Assisted Reproductive Medicine.”

7 D. Atighetchi, *Islamic Bioethics: Problems and Perspectives* (Springer, 2007).

8 H. Chamsi-Pasha and M. A. Albar, “Assisted Reproductive Technology: Islamic Sunni Perspective,” *Human Fertility* 18:2 (2015), 107–112.

9 Atighetchi, *Islamic Bioethics*.

10 Saniei and Baharvand, “Human Embryonic Stem Cell Science in Muslim Context.”

11 M. Saniei, “Human Embryo Research and Islamic Bioethics: A View from Iran,” in *Human Medical Research: Ethical, Legal and Socio-Cultural Aspects*, eds. J. Schildmann, V. Sandow, O. Rauprich and J. Vollmann (Basel: Springer, 2012), 29–41.

12 D. M. Tober and D. Budiani, “Introduction: Why Islam Health and Body?” *Body and Society* 13:3 (2014), 1–13; D. M. Tober, M. H. Taghdisi and M. Jalali, “‘Fewer Children, Better Life’ or ‘As Many as God Wants’?” *Medical Anthropology Quarterly* 20:1 (2007), 50–71.

13 M. Saniei and H. Baharvand, “La Science des Cellules Souches Embryonnaires Humaines en Iran: Une Expérience Pionnière Dans le Monde Musulman (Human Embryonic Stem Cell Science in Iran: Pioneering in the Muslim World),” *HEGEL* 4:1 (2014), 28–36.

14 M. Saniei, “Human Embryonic Stem Cell Research in Iran: The Role of the Islamic Context,” *SCRIPTed* 7:2 (2010), 324–334.

15 A. A. Alaro, “Assisted Reproductive Technology (ART): The Islamic Law Perspective,” in *Islam and Bioethics*, eds. B. Arda and R. C. Vardit (Ankara University, 2012), 95–108.

16 The holy *Qur’an*. Surah An-Nisā’ 4:1.

17 D. P. Burma and M. Chakravorty, *From Physiology and Chemistry to Biochemistry* (Pearson Education India, 2011).

18 H. E. Fadel, “The Islamic Viewpoint on New Assisted Reproductive Technologies,” *Fordham Urban Law Journal* 30 (2002), 147–157.

19 Chamsi-Pasha and Albar, “Assisted Reproductive Technology.”

20 K. A. Harwood, “On the Ethics of Social Egg Freezing and Fertility Preservation for Non-Medical Reasons,” *Medicolegal and Bioethics* 5 (2015), 59–67.

21 M. Saniei, “Human Embryonic Stem Cell Science and Policy: The Case of Iran,” *Social Science & Medicine* 98 (2013), 345–350.

22 Saniei, “Human Embryo Research and Islamic Bioethics,” 29–41; H. E. Fadel, “Developments in Stem Cell Research and Therapeutic Cloning: Islamic Ethical Positions, a Review,” *Bioethics* 26:3 (2012), 128–135.

23 M. Saniei, “Human Embryonic Stem Cell Research in Iran: The Significance of the Islamic Context,” in *Islam and Assisted Reproductive Technologies: Sunni and Shia Perspectives*, eds. M. C. Inhorn and S. Tremayne (New York: Berghahn Books, 2012), 194–219.

24 Fadel, “Developments in Stem Cell Research and Therapeutic Cloning.”

25 Atighetchi, *Islamic Bioethics*.

26 M. C. Inhorn, “Globalisation and Gametes: Reproductive ‘Tourism,’ Islamic Bioethics, and Middle Eastern Modernity,” *Anthropology & Medicine* 18 (2011), 87–103.

27 Inhorn, “Globalisation and Gametes.”

28 Chamsi-Pasha and Albar, “Assisted Reproductive Technology.”

29 P. Saxena, A. Mishra and S. Malik, “Surrogacy: Ethical and Legal Issues,” *Indian Journal of Community Medicine* 37:4 (2012), 211–213.

30 Fadel, “Developments in Stem Cell Research and Therapeutic Cloning.”

31 Saxena, Mishra and Malik, “Surrogacy.”

32 Saniei, “Human Embryonic Stem Cell Research in Iran: The Significance of the Islamic Context.”

33 S. Tremayne and M. M. Akhondi, “Conceiving IVF in Iran,” *Reproductive Biomedicine & Society Online* 2 (2016), 62–70.

34 M. Clarke, “Children of the Revolution: Ali Khamenei’s ‘Liberal’ Views on in vitro Fertilisation,” *British Journal of Middle Eastern Studies* 34:3 (2007), 287–303.

35 Ibid.

36 Tremayne and Akhondi, “Conceiving IVF in Iran”; Clarke, “Children of the Revolution.”

37 Tremayne and Akhondi, “Conceiving IVF in Iran.”

38 M. Clarke, *Islam and New Kinship: Reproductive Technology and the Shariah in Lebanon* (Berghahn Books, 2011).

39 Clarke, “Children of the Revolution.”

40 S. Jesudason and S. Baruch, “Sex Selection: What Role for Providers,” *Contraception* 86 (2012), 597–599.

41 Jesudason and Baruch, “Sex Selection.”

42 J. B. Nie, “Non-Medical Sex-Selective Abortion in China: Ethical and Public Policy Issues in the Context of 40 Million Missing Females,” *British Medical Bulletin* 98 (2011), 7–20.

43 H. Strange, “Non-Medical Sex Selection: Ethical Issues,” *British Medical Bulletin* 94:1 (2010), 7–20; I. de Melo-Martín, “Sex Selection and the Procreative Liberty Framework,” *Kennedy Institute of Ethics Journal* 23 (2013), 1–18.

44 A. M. Whittaker, “Reproduction Opportunists in the New Global Sex Trade: PGD and Non-Medical Sex Selection,” *Reproductive BioMedicine Online* 23 (2011), 609–617.

45 The holy *Qur’an*, Surah Ash-Shuraa, 42:49.

46 The holy *Qur’an*, Surah At-Takwir, 81:8–9.

47 G. I. Serour, “Ethical Issues in Human Reproduction: Islamic Perspectives,” *Gynaecological Endocrinology* 29 (2013), 949–952.

